# Procalcitonin and C-reactive protein as biomarkers for diagnosing and assessing the severity of acute cholecystitis

**DOI:** 10.1515/med-2025-1258

**Published:** 2025-09-11

**Authors:** Qisheng Hou, Huayu Li, Cheng Liu, Yujia Sun, Bo Wang, Hui Xiong, Si Liu

**Affiliations:** Department of Emergency, Peking University First Hospital, Beijing, 100034, China

**Keywords:** acute cholecystitis, Tokyo guidelines, procalcitonin, diagnosis, severity grading

## Abstract

**Background:**

Acute cholecystitis (AC) is a common condition in emergency departments (EDs), where timely diagnosis and severity assessment are critical for treatment decisions and outcome prediction.

**Methods:**

This study included 194 patients with AC to evaluate the diagnostic and severity-grading performance of procalcitonin (PCT) and C-reactive protein (CRP). Receiver operating characteristic (ROC) curve analysis was used to determine optimal cut-off values for discriminating between severity grades according to the Tokyo Guidelines.

**Results:**

Elevated levels of PCT and CRP were observed in 73.2 and 67% of cases, respectively. PCT demonstrated a sensitivity of 75.6% and specificity of 64.8% at a cut-off value of ≤ 0.595 ng/mL for distinguishing grade I from grades II to III. For differentiating grade III from grades I to II, PCT showed a sensitivity of 62.5% and specificity of 92.1% at a cut-off value of ≥5.095 ng/mL. Similarly, CRP had a sensitivity of 82.9% and specificity of 64.8% at a cut-off value of ≤78 mg/L for grade I versus grades II to III, and a sensitivity of 62.5% and specificity of 69.7% at a cut-off value of ≥82.5 mg/L for grade III versus grades I to II.

**Conclusion:**

Both PCT and CRP are valuable biomarkers for diagnosing AC and assessing its severity.

## 1 Introduction

Acute cholecystitis (AC) frequently presents in emergency departments (EDs). The typical presentation of AC consists of acute right upper quadrant (RUQ) pain, fever, and nausea that may be associated with eating and physical examination findings of RUQ tenderness. Its occurrence is often related to gallstone obstruction of the cystic duct, release of inflammatory mediators, and bile infection in the biliary system [1–5].

The Tokyo Guidelines [6] established criteria for diagnosing and grading the severity of AC, aiming to reduce mortality and morbidity in patients with AC and to provide the most appropriate treatment plan at an early stage. Laboratory tests such as white blood cell (WBC) counts and C-reactive protein (CRP) levels are used for diagnosing AC, with increased WBC counts being related to the severity of AC. However, the value of procalcitonin (PCT), which is highly specific for infection [7,8], in diagnosing and grading the severity of AC is currently unclear, as is the value of CRP in grading the severity of AC.

The aim of this study was to evaluate the potential value of PCT and CRP in diagnosing and grading the severity of AC. We hypothesized that PCT and CRP would be helpful in diagnosing and grading the severity of AC.

## 2 Methods

### 2.1 Study design

This retrospective study was conducted in the ED of Peking University First Hospital, which provides round-the-clock emergency medical services and treats approximately 120,000 patients annually.

This study was approved by the Biomedical Research Ethics Committee of Peking University First Hospital, and all methods were performed in accordance with the relevant guidelines and regulations.

### 2.2 Participants

The medical records of all patients diagnosed with AC admitted to the ED of Peking University First Hospital from September 1, 2021 to August 30, 2022 were retrospectively collected. Inclusion criteria were participants must meet the diagnostic criteria outlined in the Tokyo Guidelines 2018 for AC and eligible subjects must be 18 years of age or older. Exclusion criteria were participants with concurrent infectious diseases that may influence PCT and CRP results (such as acute cholangitis or pneumonia) and the results of PCT or CRP were not recorded.

The ED of Peking University First Hospital routinely performed complete blood counts, CRP, liver and kidney function tests, coagulation function tests, and PCT tests for patients with suspected AC who present with the chief complaint of “RUQ pain,” and meanwhile performed B-ultrasound or computed tomography (CT) to confirm the diagnosis. Blood sampling for inflammatory indicators was taken before the application of antibiotics. Clinicians in the ED determined the necessity of performing an arterial blood gas analysis and the administration of intravenous fluids, vasoactive medications such as dopamine or norepinephrine to maintain blood pressure, or oxygen therapy based on the patient’s hemodynamic parameters, including blood pressure, heart rate, level of consciousness, respiratory status, and oxygen saturation. All diagnostic tests and clinical management strategies were conducted within the ED, ensuring a thorough and coordinated approach to the care of patients with suspected AC.

Participants had serum or plasma collected for PCT measurement within 2 h after admission using the Vidas BRAHMS PCT according to manufacturer instructions (bioMérieux, Marcy-l’Étoile, France).

### 2.3 Data collection and analysis

The gender, age, onset time of abdominal pain, RUQ pain, fever (>37.3°C), RUQ tenderness, RUQ mass, Murphy’s sign, cardiovascular dysfunction, neurological dysfunction, respiratory dysfunction, renal dysfunction, hepatic dysfunction, hematological dysfunction, WBC, CRP, platelet count, serum creatinine, international normalized ratio (INR), PCT, B-ultrasound, or CT diagnosis of all subjects were collected and recorded. And whether PCT (normal reference values: <0.05 ng/mL), WBC (normal reference values: 3.5–9.5 × 10^9^/L), and CRP (normal reference values: 0–8 mg/L) were abnormal were also collected and recorded.

The proportion of elevated PCT, WBC, and CRP in all patients with AC was evaluated. Patients were classified into three stages, namely, grade I, grade II, and grade III, according to the severity grading of AC using the Tokyo Guidelines 2018. Laparoscopic cholecystectomy is routinely recommended for patients classified as grade I. For patients with grade II, either laparoscopic cholecystectomy or percutaneous transhepatic gallbladder drainage may be considered as appropriate management options. In cases of grade III, percutaneous transhepatic gallbladder drainage is generally recommended. However, treatment decisions may also take into account the preferences of the patient and their family. In such instances, a conservative approach based on antibiotic therapy, analgesia, and fluid resuscitation may be adopted when indicated. The role of PCT and CRP in the assessment of severity of AC and the correlation between the stages and PCT and CRP were statistically analyzed.

### 2.4 Statistical analysis

Statistical analyses were performed using IBM SPSS Statistics version 27 (IBM Corporation, Armonk, NY, USA). Numerical data were assessed for normality using the single-sample Kolmogorov–Smirnov test. Data conforming to a normal distribution were expressed as mean values with standard deviation (±SD). For comparisons between independent sample groups, one-way analysis of variance was employed. Non-normally distributed numerical data were represented as median values with interquartile ranges [*M* (*Q*1, *Q3*)]. The Kruskal–Wallis *H-*test was utilized for comparisons among independent sample groups with non-normal distributions. The multivariate logistic regression analysis was used to determine the predictors that could influence the increased levels of the studied parameters. Spearman’s correlation analysis was applied to evaluate the direction and strength of relationships between variables that did not adhere to a normal distribution. Categorical data were presented as percentages and were compared using the chi-squared test or Fisher’s exact test, as appropriate. A *p*-value of less than 0.05 was considered to indicate statistical significance. The optimal cut-off value for test validity, as well as sensitivity and specificity, were determined through receiver operating characteristic (ROC) analysis.


**Ethics approval:** This study was approved by the Biomedical Research Ethics Committee of Peking University First Hospital, and the registration number is 2022 224-001.

## 3 Results

### 3.1 Participants and basic data

**Figure 1:**
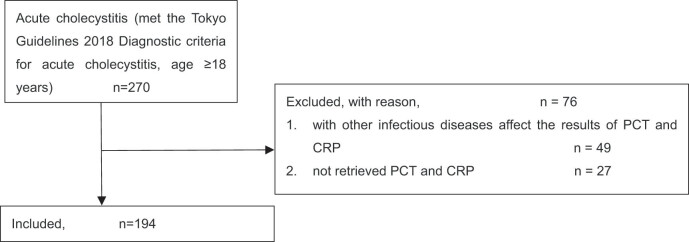
Patient enrolment flow chart.

A total of 270 patients over the age of 18 met the Tokyo guidelines 2018 diagnostic criteria of AC, 76 patients with other infectious diseases or no PCT or CRP results were excluded, and finally 194 patients were included in this study (Figure 1). All patients with AC were diagnosed by B-ultrasound or CT. The demographic data, basic data, and comparison between groups after grouping all participants according to the Tokyo Guidelines 2018 severity grading criteria of AC are shown in Table 1.

**Table 1:** Demographic data, basic data, and comparison between groups

	Total	Grade I	Grade II	Grade III	Statistical value	*P* value
	*n* = 194	*n* = 123	*n* = 55	*n* = 16		
Gender					*X* ^2^ = 2.589	0.274
Male	113 (58.2)	67 (54.5)	37 (67.3)	9 (56.3)		
Female	81 (41.8)	56 (45.5)	18 (32.7)	7 (43.7)		
Age (years)	67 (56.75, 76)	65 (55, 74)	69 (53, 82)	72.5 (62, 85)	*H* = 5.886	0.053
Onset time of abdominal pain (h)	24 (9.75, 72)	24 (6, 24)	96 (48, 144)	24 (3, 96)	*H* = 66.898	<0.001
Fever					*X* ^2^ = 13.533	0.001
+	58 (29.9)	26 (21.1)	23 (41.8)	9 (56.3)		
−	136 (70.1)	97 (78.9)	32 (58.2)	7 (43.7)		
RUQ tenderness					*X* ^2^ = 1.229	0.541
+	160 (82.5)	99 (80.5)	48 (87.3)	13 (81.3)		
−	34 (17.5)	24 (19.5)	7 (12.7)	3 (18.7)		
Murphy’s sign					*X* ^2^ = 0.382	0.826
+	61 (31.4)	40 (32.5)	17 (30.9)	4 (25)		
−	133 (68.6)	83 (67.5)	38 (69.1)	12 (75)		
WBC (×10^9^/L)	11.205 (8.9, 14.225)	11.05 (8.74, 13.5)	12.46 (10.1, 19.04)	10.405 (6.6, 12.425)	*H* = 10.183	0.006
CRP (mg/L)	25 (3, 103)	9 (1, 56)	103 (34, 177)	94.5 (17.5, 254.75)	*H* = 48.308	<0.001
Platelet count (×10^9^/L)	222.02 ± 74.475	221.58 ± 69.632	235.73 ± 72.669	178.25 ± 100.463	*F* = 3.806	0.024
Serum creatinine (μmol/L)	84.2 (72.625, 101.175)	82.4 (70.75, 95.05)	86.1 (74.3, 104.6)	166.25 (83.2, 272.6)	*H* = 17.453	<0.001
INR	1.02 (0.94, 1.115)	0.97 (0.93, 1.05)	1.09 (1.02, 1.16)	1.04 (0.95, 1.22)	*H* = 22.722	<0.001
PCT (ng/mL)	0.345 (0.04, 1.6725)	0.11 (0.04, 0.57)	0.85 (0.12, 3.52)	8.685 (0.58, 21.38)	*H* = 40.238	<0.001
PCT					*X* ^2^ = 22.297	<0.001
+	142 (73.2)	76 (61.8)	51 (92.7)	15 (93.8)		
−	52 (26.8)	47 (38.2)	4 (7.3)	1 (6.2)		
WBC					*X* ^2^ = 3.659	0.161
+	143 (73.7)	90 (73.2)	44 (80)	9 (56.3)		
−	51 (26.3)	33 (26.8)	11 (20)	7 (43.7)		
CRP					*X* ^2^ = 30.549	<0.001
+	130 (67)	65 (52.8)	50 (90.9)	15 (93.8)		
−	64 (33)	58 (47.2)	5 (9.1)	1 (6.2)		

Note: The statistical values and *P*-values presented in the table represent the results of comparisons among different groups categorized by severity grades (Grade I, Grade II, and Grade III).

Among 194 patients with AC, PCT, WBC, and CRP elevated in 73.2% (95% confidence interval [CI] 67–79%), 73.7% (95% CI 68–80%), 67% (95% CI 60–74%) (Table 1).

### 3.2 Correlation analysis

The onset time of abdominal pain, fever, WBC, CRP, platelet count, serum creatinine, INR, and PCT were related to the severity of AC, and the differences were statistically significant (Table 1). The results of the multivariate logistic regression analysis are shown in Table 2. Among them, the onset time of abdominal pain, WBC, platelet count, serum creatinine, and INR are part of the reference criteria for the severity grading of AC according to the Tokyo Guidelines 2018. The relationship between the above indicators and the severity grading of AC is not further analyzed in this study.

**Table 2:** Results of multivariate logistic regression analysis

Severity grading		*B*	Standard error	*P*
Grade I	Onset time of abdominal pain	−0.056	0.012	<0.001
	Fever	−1.810	0.892	0.042
	WBC	−0.226	0.072	0.002
	CRP	−0.012	0.005	0.025
	Platelet count	−0.007	0.005	0.145
	Serum creatinine	−0.033	0.014	0.016
	INR	3.462	2.134	0.105
	PCT	−0.061	0.048	0.206
Grade III	Onset time of abdominal pain	0.007	0.007	0.331
	Fever	1.616	1.416	0.254
	WBC	−0.094	0.098	0.338
	CRP	−0.007	0.006	0.249
	Platelet count	−0.012	0.009	0.201
	Serum creatinine	0.038	0.013	0.004
	INR	1.287	2.598	0.620
	PCT	0.131	0.052	0.011

Note: The reference category was Grade II.

CRP and PCT were significantly correlated with the severity grading of AC, and the higher the CRP and PCT values, the higher the severity grading (Table 3).

**Table 3:** Correlation analysis between CRP, PCT, and severity grading of AC

	Severity grading of AC	
CRP	*r* = 0.489	*P* < 0.001
PCT	*r* = 0.452	*P* < 0.001

Note: Spearman correlation analysis.

### 3.3 ROC analysis

**Figure 2:**
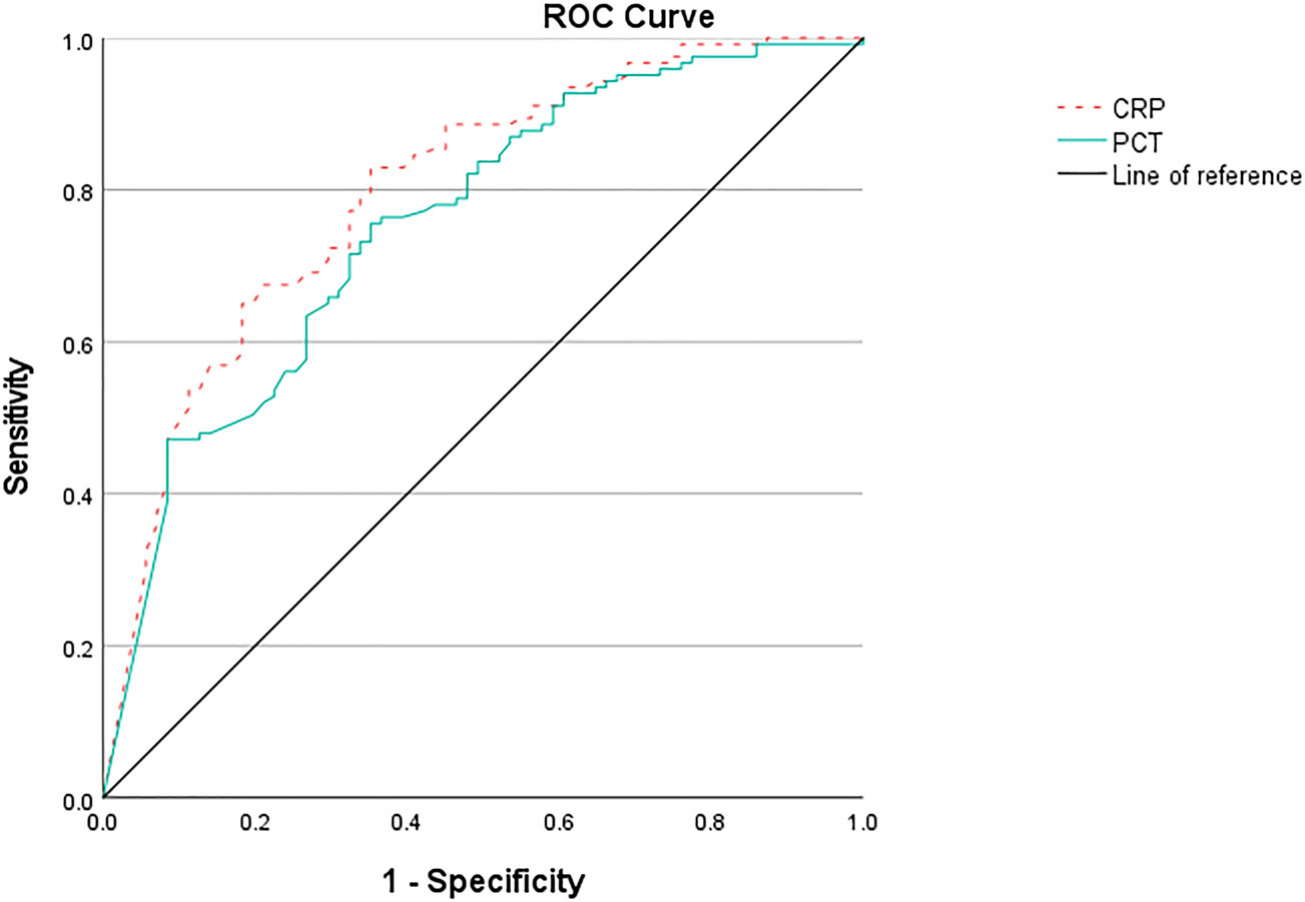
ROC curve of discriminate grade I from grade II to III.

According to the ROC curve (Figure 2), when grade II and grade III groups were combined as the control group, PCT could discriminate grade I from grade II to III with 75.6% sensitivity and 64.8% specificity at the best cut-off value of ≤0.595 ng/mL, the area under the curve (AUC) was 0.756 (95% CI 0.685–0.826), the Youden index was 0.404, the positive predictive value (PPV) was 78.7%, the negative predictive value (NPV) was 60.6%, and the accuracy was 71.6%. CRP could discriminate grade I from grade II to III with 82.9% sensitivity and 64.8% specificity at the best cut-off value of ≤78 mg/L, the AUC was 0.798 (95% CI 0.733–0.863), the Youden index was 0.477, the PPV was 80.3%, the NPV was 68.7%, and the accuracy was 76.2%.

**Figure 3:**
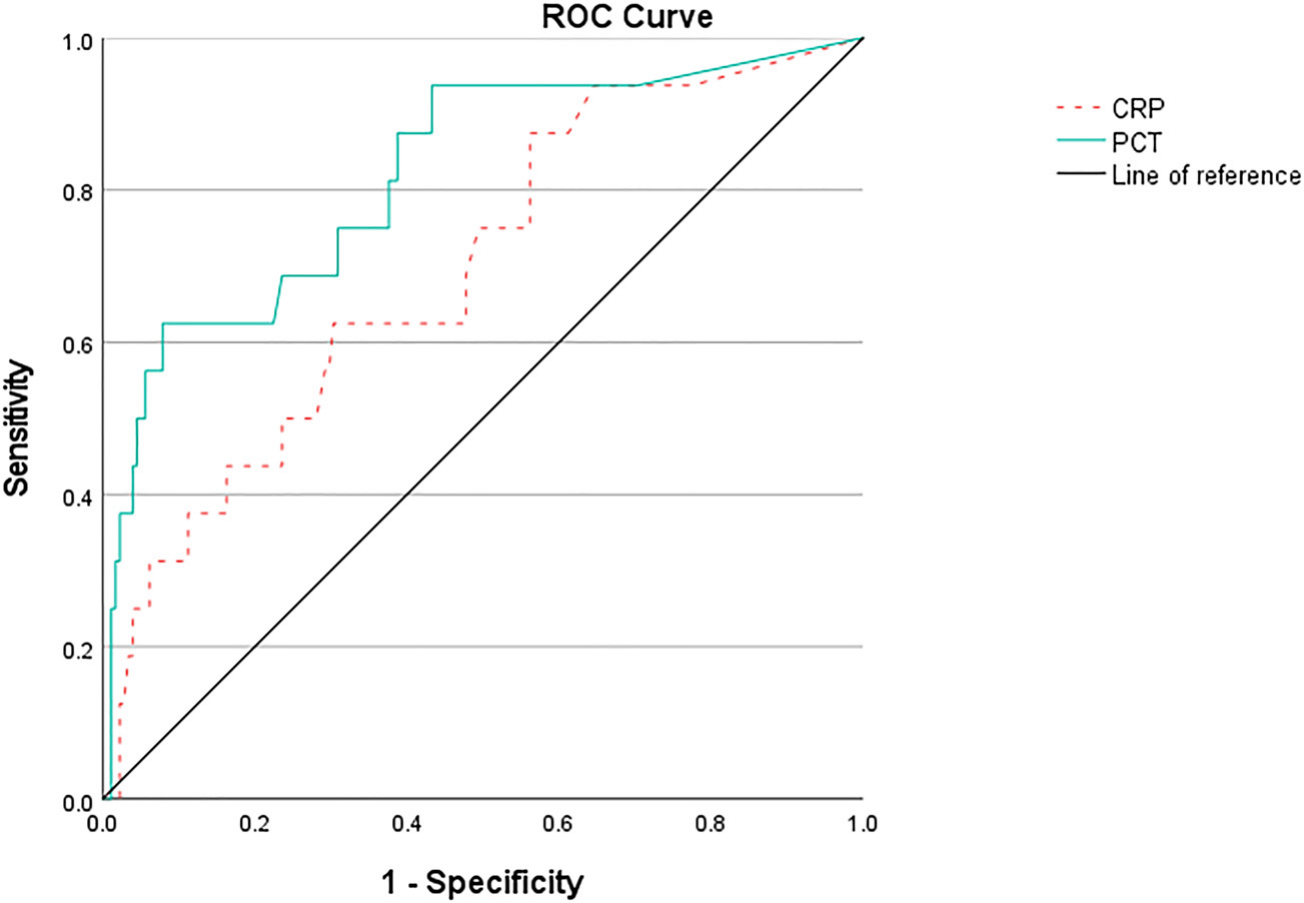
ROC curve of discriminate grade III from grade I to II.

According to the ROC curve (Figure 3), when grade I and grade II groups were combined as the control group, PCT could discriminate grade III from grade I to II with 62.5% sensitivity and 92.1% specificity at the best cut-off value of ≥5.095 ng/mL, the AUC was 0.819 (95% CI 0.703–0.936), the Youden index was 0.546, the PPV was 41.4%, the NPV was 96.5%, and the accuracy was 89.6%. CRP could discriminate grade III from grade I to II with 62.5% sensitivity and 69.7% specificity at the best cut-off value of ≥82.5 mg/L, the AUC was 0.695 (95% CI 0.562–0.828), the Youden index was 0.322, the PPV was 15.6%, the NPV was 95.4%, and the accuracy was 69.1%.

## 4 Discussion

### 4.1 Value of PCT in diagnosis of AC

AC is a common disease in the ED. As no feature has sufficient diagnostic power to establish or exclude the diagnosis of AC, it is recommended not to rely on a single clinical or laboratory finding. For the diagnosis of AC, it is suggested using a combination of detailed history, complete clinical examination, laboratory tests, and imaging investigations [1,9]. The Tokyo Guidelines 2018 diagnostic criteria for AC [6] should meet three aspects: local signs of inflammatory, systemic signs of inflammatory, and imaging findings. Fever, elevated CRP, and elevated WBC count are specific reference indicators of systemic signs of inflammatory. In this study, PCT, WBC, and CRP elevated in 73.2% (95% CI 67–79%), 73.7% (95% CI 68–80%), 67% (95% CI 60–74%), respectively, among all patients diagnosed with AC, indicating that the diagnostic value of PCT is equivalent to that of CRP and WBC. It has also been found in previous literature that PCT can increase rapidly within the first hours after systemic inflammation and peaked earlier than CRP, while CRP is usually within the normal range in the first 6–12 h of the onset of AC [10–12]. It revealed that PCT may be helpful for the early diagnosis and identification of AC with infection or systemic inflammation.

### 4.2 Value of PCT in grading severity of AC

After early and accurate diagnosis of AC, timely and correct intervention is very important to reduce the mortality and complication rate of AC. Grading the severity of AC can help predict the outcome of patients with AC and guide treatment decisions [6,9,13]. According to the Tokyo Guidelines 2018 [6], AC is divided into three grades (grade I, grade II, and grade III) according to the presence or absence of organ or system dysfunction, the degree of systemic and local inflammatory response, duration of onset, etc. According to the severity grading, Charlson comorbidity index and American Society of Anesthesiologists physical status classification, the timing of laparoscopic cholecystectomy and the conditions required for surgery in patients with AC were guided [13]. The value of PCT in the severity grading of AC was not clear.

In this study, it was shown that there was a significant correlation between PCT and the severity grading of AC (*r* = 0.452, *P* < 0.001). As the severity grading increased, the PCT levels were found to be statistically significantly elevated (*H* = 40.328, *P* < 0.001). Taking PCT ≤ 0.595 ng/mL as the diagnostic cut-off value, the sensitivity, specificity, and accuracy of PCT in distinguishing grade I from grade II to III AC were, respectively, 75.6, 64.8, and 71.6%. Taking PCT ≥ 5.095 ng/mL as the diagnostic cut-off value, the sensitivity, specificity, and accuracy of distinguishing grade III from grade I to II AC were 62.5, 92.1, 89.6%, respectively. The results showed that PCT had a good value in grading the severity of AC.

Yuzbasioglu et al. [12] investigated the relationship between PCT level and the severity grading in 200 patients with AC and found that PCT level was helpful for severity grading of AC, and it was suggested that PCT level may be considered to be a parameter that would be added to the assessment of the severity grading of AC in the Tokyo Guidelines. However, in Yuzbasioglu’s study, AC was diagnosed based on the clinical suspicion and at least one of the local or systemic inflammatory findings, combined with ultrasonographic imaging. According to the diagnostic criteria of Tokyo Guidelines 2018 [6] for AC, local signs of inflammation, systemic signs of inflammation, and imaging findings should be met at the same time to diagnose AC definitely. In this study, the diagnostic criteria of AC in Tokyo Guideline 2018 [6] were used as one of the inclusion criteria, which was stricter and more accurate. Sakalar et al. [14] also found that PCT levels could be used to determine the severity of AC effectively in their study of 95 patients with AC, but PCT was measured after the clinical and radiological diagnosis of AC rather than before, and the sample size was relatively small. In this study, PCT was collected at the same time as other laboratory tests such as WBC and CRP before the diagnosis of AC, and the sample size was larger, the results were more representative.

Fransvea et al. [15] conducted a retrospective study on the preoperative PCT results of 174 patients with acute calculous cholecystitis who underwent laparoscopic cholecystectomy and found that PCT > 0.09 ng/mL was associated with a poor surgical outcome for acute calculous cholecystitis. Wu et al. [16] conducted a retrospective study on 115 patients with AC who underwent laparoscopic cholecystectomy and found that PCT > 1.5 ng/mL was associated with difficult laparoscopic cholecystectomy. In these studies, patients with AC who underwent laparoscopic cholecystectomy were analyzed, and the value of PCT in patients with AC who did not undergo laparoscopic cholecystectomy could not be evaluated. In this study, participants were patients with AC who met the diagnostic criteria of Tokyo Guidelines 2018, regardless of whether they had surgery or not. The participants were more comprehensive and specific.

The results of this study and previous studies suggest that PCT is helpful to evaluate the severity of AC, the higher the PCT, the more severe the severity, and PCT has an impact on the difficulty of surgery and poor prognosis of patients. It is helpful to guide the treatment decision and predict the outcomes of patients with AC when the PCT level is included as one of the reference indicators for the severity grading of AC.

### 4.3 Value of CRP in grading severity of AC

In the Tokyo Guidelines 2018 [6], CRP and WBC count are the only two laboratory tests in the diagnostic criteria for AC, but only WBC count is the reference index in the severity grading of AC. The value of CRP for AC severity grading is unclear.

In this study, it was shown that there was a significant correlation between CRP and the severity grading of AC (*r* = 0.489, *P* < 0.001). As the severity grading increased, the CRP levels were found to be statistically significantly elevated (*H* = 48.308, *P* < 0.001). Taking CRP ≤ 78 mg/L as the diagnostic cut-off value, the sensitivity, specificity, and accuracy of CRP in distinguishing grade I from grade II to III AC were, respectively, 82.9, 64.8, 76.2%. The diagnostic accuracy of CRP was similar to that of PCT (76.2 vs 71.6%). Taking CRP ≥ 82.5 mg/L as the diagnostic cut-off value, the sensitivity, specificity, and accuracy of distinguishing grade III from grade I to II AC were 62.5, 69.7, 69.1%, respectively. The diagnostic accuracy of CRP was lower than that of PCT (69.1 vs 89.6%). The results showed that CRP had a good value in grading the severity of AC. The value of CRP in distinguishing grade I from grade II to III was higher than that in distinguishing grade III from grade I to II. A prospective cohort study of 556 subjects [17] also found that CRP is the best inflammatory marker for predictive of advanced AC (gangrenous cholecystitis, pericholecystic abscess, hepatic abscess, biliary peritonitis, emphysematous cholecystitis) and of conversion to open surgery. It was suggested that CRP should be considered as a severity criterion of AC in the Tokyo Guidelines.

## 5 Limitation

This study was a single-center and retrospective analysis, and there is no guarantee of the completeness of the recorded patient data. The number of patients with grade III AC is small, and the results may have some bias. Second, although our center routinely performed the examination of PCT and CRP for patients suspected of AC with the chief complaint of “RUQ pain,” there were still some patients who did not perform the examination of PCT or CRP or could not retrieve the corresponding data, which affected the integrity of the data. The multi-center, prospective, large sample size study is helpful to further evaluate the value of PCT and CRP in the diagnosis and severity grading of AC.

## Abbreviations

AC: acute cholecystitis
AUC: area under the curve
CI: confidence interval
CRP: C-reactive protein
CT: computed tomography
EDs: emergency departments
INR: international normalized ratio
NPV: negative predictive value
PCT: procalcitonin
PPV: positive predictive value
ROC: receiver operating characteristic
RUQ: right upper quadrant
SPSS: Statistical Product Service Solutions
WBC: white blood cell
